# Hard X-ray nanotomography reveals anomalous and expected thermal coarsening behaviour of nanoporous gold

**DOI:** 10.1039/d5na00561b

**Published:** 2025-11-20

**Authors:** Reihaneh Pashminehazar, Yakub Fam, Ana Diaz, Mirko Holler, Michal Kronenberg, Johannes Ihli, Jan-Dierk Grunwaldt, Thomas L. Sheppard

**Affiliations:** a Institute for Chemical Technology and Polymer Chemistry, Karlsruhe Institute of Technology Engesserstrasse 20 76131 Karlsruhe Germany thomas.sheppard@tuwien.ac.at grunwaldt@kit.edu; b Institute of Catalysis Research and Technology, Karlsruhe Institute of Technology Hermann-von-Helmholtz Platz 1 76344 Eggenstein-Leopoldshafen Germany; c PSI Center for Photon Science, Paul Scherrer Institut Forschungsstrasse 111 5232 Villigen PSI Switzerland

## Abstract

Ptychographic X-ray computed tomography was used to image nanoporous gold samples, with and without metal oxide additives, following incremental *ex situ* annealing steps up to 750 °C. Studying the exact same sample volumes following sequential annealing steps allowed accurate 3D imaging of large meso- and macropore systems over extended sample volumes. Extraction of surface area, pore size distribution, and pore connectivity were demonstrated using a skeletonization method. These properties are relevant in the study of functional materials such as catalysts which rely on diffusion processes within the pores. Samples with metal oxide additives were found to be more resistant to thermal annealing and gold ligament coarsening up to 550 °C, while pure nanoporous gold showed a greater loss of specific surface area during the same treatment. An anomalous stabilisation effect was observed during measurements in ambient air, with minimal coarsening observed in sample regions previously exposed to X-rays, and extensive coarsening in neighbouring regions of the same sample which were not previously exposed to X-rays. Thermal annealing of duplicate samples under nitrogen flow eliminated this effect, suggesting the possible formation of a protective surface structure induced by X-ray irradiation of nanoporous gold in air. The same observations may not be visible to conventional bulk sorption or porosimetry methods, showing the benefits of X-ray tomography for quantitative spatially-resolved imaging of porous nanomaterials.

## Abstract 2

X-ray nanotomography reveals pore structure changes in 3D space and stabilisation effects towards thermal annealing.

## Graphical abstract

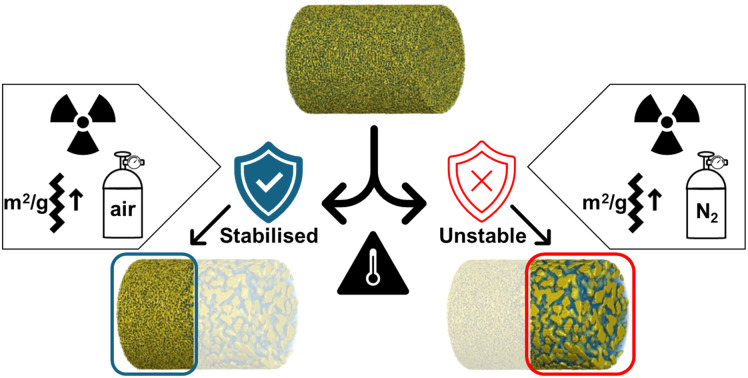

## 1. Introduction

In heterogeneous catalysis, material function is closely related to sample structure and chemical environment across multiple length scales.^1–4^ A detailed understanding of catalytic properties ideally requires multiscale structural studies targeted to the features of interest. For example, porosity and the structure of pore networks are important topics in terms of diffusion, transport phenomena, and mechanical stability.^3–7^ In typical solid catalysts with active metal species distributed in the pores, the internal pore network determines accessibility of reactants and diffusion of gas or liquid molecules, therefore determining catalyst performance. Commonly applied methods to study pore systems include combinations of gas sorption studies (*e.g.* N_2_, Ar) and/or porosimetry methods (*e.g.* mercury intrusion).^2,8,9^ Although fast and convenient, such bulk porosimetry methods typically provide only numerically averaged data and do not provide localized data on individual pores. Furthermore, such methods are not sensitive to finer structural features such as pore geometry, tortuosity, or branching. As a result, conventional porosimetry methods can be reasonably applied to interpret the average composition of a sample, but quantifying the structure of more complex systems such as hierarchically porous materials can be challenging. The latter are often used in technical and industrial catalysis.^10–12^

In comparison to conventional pore characterization methods, tomography particularly with hard X-rays has demonstrated excellent potential for the study of porous materials.^13,14^ Tomography allows non-invasive 3D spatially-resolved imaging of the sample. In particular, the study of hierarchical pore systems can benefit greatly from the statistical analysis of pores enabled by tomography. Regardless of the specific probe used, tomography studies must compromise between the spatial resolution desired, and the sample size which can be studied within a given time duration (*e.g.* typically a synchrotron beamtime allocation).^3,15^ Compared to alternative probes such as electron tomography,^15–17^ or focused ion beam serial sectioning,^18–20^ methods such as ptychographic X-ray computed tomography (PXCT), holotomography, and transmission X-ray tomography, have shown excellent performance, balancing high spatial resolution and representative sample volume.^4,7,21–24^ Achieving a large number of resolution elements over extended length scales is particularly important to ensure measurement of structurally representative sample volumes, particularly for complex hierarchical or composite materials. The disadvantage of hard X-ray tomography collectively is that these methods require access to specialized equipment or large research facilities such as synchrotron radiation sources.

The continuing development of fourth generation synchrotron light sources is expected to revolutionize the field of hard X-ray imaging, particularly for high-resolution coherence methods such as PXCT and holotomography,^25–28^ which are already known for achieving exceptional 3D spatial resolution of extended sample volumes. Previously, Larsson *et al.*^29^ proposed the concept of using hierarchical nanoporous gold (np-Au) as a multiscale 3D test pattern for characterizing hard X-ray nanotomography setups. As np-Au contains a hierarchical system of pores at multiple length scales in a disorganized or stochastic arrangement,^30^ this is in some sense a 3D analogue to the common “Siemens star” as a 2D resolution standard. Larsson *et al.* compared full-field transmission X-ray tomography with a previous study from our group based on PXCT,^19^ obtaining consistent results concerning sample porosity within the observed resolution limits of 50 nm and 23 nm respectively.^29^

Here we present PXCT of a np-Au sample series with 3D spatial resolution of *ca.* 10–20 nm, which is possible due to the excellent contrast between gold ligaments and surrounding air. The exceptional resolution obtained allows to extend our previous work to compare compositional variants of np-Au with the inclusion of metal oxide additives (TiO_2_ and CeO_2_), which have been proposed to increase the resistance of np-Au towards thermal annealing.^31,32^ Furthermore, we demonstrate sequential correlative analysis of the exact same sample regions at different stages of thermal annealing, allowing comparison of annealing effects depending on the sample composition. This also allowed the observation of anomalous stabilization effects depending on annealing atmosphere (air or N_2_), and previous exposure to X-rays.

A typical workflow is presented for analyzing the pore structures based on the volumetric tomography data obtained. In addition to high-resolution tomography, quantitative analysis of the pore systems proves to be challenging for such highly porous materials.^33–35^ A defining characteristic of all porous materials is that the pore structure can significantly impact transport processes. Even with identical percentage porosity, two materials may exhibit vastly different transport properties due to factors like the spatial distribution of pores, connectivity, shape, or size distribution for example.^36,37^ Various methods are available to analyze the pore system in greater detail. Two key algorithms in this context are the medial axis-based method and the maximal ball (MB) method.^33,36,38,39^ In this study, porosity, surface area, and pore connectivity were analyzed using the skeletonization method, which is based on the medial axis algorithm. This method enables structural simplification to aid with the analysis while retaining essential features of the original pore network shape, which typically emphasizes geometrical and topological properties. Relevant pore features are therefore discussed for np-Au samples before and after annealing.

## 2. Methods

### 2.1. Sample preparation

Pure np-Au samples and np-Au incorporated with CeO_2_ (denoted CeO_*x*_/np-Au) and a combination of CeO_2_ and TiO_2_ (denoted Ce-TiO_*x*_/np-Au) were prepared by dealloying of an Ag–Au composite, followed by wet impregnation with metal-oxide precursor solutions using the methods described in previous reports.^32,40,41^ Pieces of each sample were placed on SEM sample holder stubs, then cut and shaped with a Ga^+^ focused ion beam (FIB) using a FIB Strata 400S (Thermo Fisher, USA) into pillars with diameters in the range of 4–6 µm and heights in the range of 10–30 µm. The prepared pillars were then transferred to a customized Cu sample holder pin (OMNY pin).^42^ We note that the standard OMNY pin has an Au coating to facilitate mounting in cryo conditions. However, for our experiments the Cu OMNY pins were not Au coated. Analogous FIB-SEM preparation procedures were detailed in Fam *et al.* (2018).^19^

### 2.2. PXCT measurements and data reconstruction

PXCT measurements were performed at the coherent small-angle X-ray scattering (cSAXS) beamline of the Swiss Light Source at the Paul Scherrer Institute (Villigen, Switzerland) with the flexible tomography nanoimaging endstation (flOMNI) setup.^43,44^ An Au-made Fresnel zone plate (FZP) with an outer-most zone width of 60 nm was employed to define a coherent illumination onto the sample, providing a flux of about 4 × 10^8^ photons per s. Most measurements were performed at a photon energy of 6.2 keV, while one of the measurements was performed at an energy of about 4.9 keV.

The sample was placed at a distance close to 1 mm downstream of the focus, so that the illumination had a diameter of a few microns on the sample. A single photon counting detector was placed in the far field at a distance of several meters. For the pure nanoporous gold sample (np-Au (1)) measurements, a Pilatus 2M detector in air was used, placed 7 m downstream of the sample with a He-filled flight tube placed in between to reduce background scattering from air. For the other samples including metal oxide additives (CeO_*x*_/np-Au, Ce-TiO_*x*_/np-Au) we used an in-vacuum Eiger 1.5M detector placed 5 m downstream of the sample. The detector was located inside an evacuated flight tube. Both detectors were developed and fabricated by the detector group at the Paul Scherrer Institute (Villigen, Switzerland). Ptychographic acquisitions were performed by scanning the sample using a modified Fermat spiral trajectory.^45^ Ptychographic scans were repeated from 0 to 180° of sample rotation for tomography. The dose absorbed by the sample was calculated for each tomographic acquisition by measuring the total number of photons absorbed by the sample during the first ptychographic scan, the measurement of the total mass of the sample provided by the quantitative 3D electron density map of the sample obtained by PXCT, and the total number of projections. In Table S1 in the SI we list all tomographic acquisitions with the sample names and their measurement conditions, while in Tables S2 and S3 we show the experimental parameters relevant for data acquisition for all the PXCT datasets.

Ptychographic scans were recorded in pairs at different tomographic angles and lateral positions of the detector, and each pair was combined under the same reconstruction and illumination, resulting in two different images.^42^ For the ptychographic image reconstruction we used a combination of difference map algorithm and maximum likelihood optimization.^46,47^ For some of the datasets, ptychographic reconstructions provided better resolution when using almost all the pixels of the Eiger 1.5M detector by using a non-squared cropped area of the detector, resulting in a non-isotropic reconstructed pixel size. In these cases, reconstructions were performed using a generalized iterative least squares solver implemented for graphics processing units (GPU), which we abbreviate MLc.^48^ After the reconstruction process, the images with anisotropic pixel size were upsampled along the vertical direction to result in an isotropic pixel size. In Table S4 we write the details of the algorithms and parameters used for each tomographic dataset. The phase images were then aligned in vertical and horizontal directions^49,50^ Tomographic reconstruction *via* filtered back projection was used to generate tomograms. For most of the tomograms, 20 iterations of the simultaneous algebraic reconstruction technique (SART) method were further used to provide better results. The 3D spatial resolution of the reconstructed 3D tomograms was determined *via* Fourier shell correlation (SI Section 5 and Table S4).^48^

Sequential thermal treatment of the samples in between PXCT measurements was carried out in ambient air using an external portable furnace (Nabertherm, DE). For the pure nanoporous gold sample (np-Au (1)), a heating rate of 10 °C min^−1^ was used until 450 °C, followed by a hold time of 1 hour. All other samples were heated at a rate of 10 °C min^−1^, first to reach a temperature of 550 °C and then 750 °C. The hold time at each temperature was 2 hours. After each PXCT measurement, the OMNY pin containing the sample was transferred from the PXCT instrument into the furnace and heated to the required temperature points as indicated in the relevant figure captions. Afterwards, the OMNY pin was transferred back to the PXCT experiment setup for further measurement.

### 2.3. Tomography image processing

**Fig. 1:**
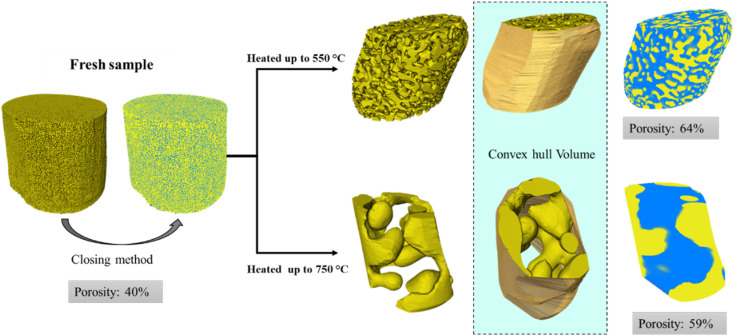
Summary of tomography data processing, including morphological closing (fresh samples), or convex hull generation and segmentation with labelling of solid and pore phases (thermally treated samples).

Tomographic image data was first treated with an edge-preserving smoothing filter, for denoising and image enhancement. A morphological closing function was then performed to fill small pores. This function consists of a dilation followed by an erosion operation, both using the same structuring element. As a result, it is less destructive than other morphological operations and preserves the original size, shape, and convexity of the analyzed structure. This method is particularly effective in defining the pore volume of samples with small pores, such as fresh np-Au samples. However, for images featuring sintered or large pores, the closing method is not applicable. Attempting to fill a large pore could lead to undesired deformation of the object's exterior surface. Therefore, to artificially limit pore volumes occurring at the exterior of the np-Au solid phase, a convex hull was generated to circumscribe the solid and produce an artificial boundary of the exterior pores. A summary of pre-analysis steps for calculating the porosity of the samples is shown in Fig. 1. Following these steps, basic characterization of sample porosity and surface area could be calculated. All 3D volume renderings of the entire sample and extracted region of interest were performed using Avizo software (ThermoFisher). Further details are given in the SI (Section 1).

### 2.4. Skeletonization and skeleton analysis

For topological analysis of the segmented pore network, skeletonization was used to generate a simplified geometrical and topological map of the pores. Skeletonization reduces all objects in the binary image to 1-pixel wide curved lines which are equidistant to its boundaries. This allows for properties such as connectivity, topology, length and direction to be calculated. For this, binary images were first generated of the solid and pores. The MATLAB function *bwskel* was then used to generate the skeleton structure of the pore system from the binarized tomography data. Finally, a pruning algorithm was used to remove unwanted and small skeleton branches to get a clean skeleton representation. The Skel2Graph3D code developed by Kollmannsberger^51^ was then used to convert the 3D binary voxel skeleton into a network graph described by nodes and branches. The input is a 3D binary image containing a one-dimensional voxel skeleton. The output is the adjacency matrix of the graph, and the nodes and links of the network as MATLAB structure. Further analysis including total length of pore network, branch size distribution, end point (blind pore) and connectivity of the pore system were done by self-developed MATLAB code. Node branch points (Bp) were defined as voxels at a junction where multiple branches meet (voxels with more than 2 neighbours), and end points (Ep) were defined as voxels at the ends of branches (voxels with less than 2 neighbours). The visual representation of the definition is available in the SI (Fig. S2). The skeleton analysis was conducted on the entire sample as well as on various sub-volumes within each sample. This approach was used to compare homogeneity between sub-volumes and therefore representativeness compared to the entire sample. The skeletonization analysis defined here was applied for all samples except those heated up to 750 °C, since sintering caused extensive degradation of the pore system after this point. Further details are given in the SI (Section 2).

### 2.5. Pore connectivity analysis

An average pore system connectivity (*Z*) was calculated as the average number of branches of the skeleton structure meeting at a junction (*i.e.* a node of the skeleton network). The following definition was used by Hormann *et al.*^52^
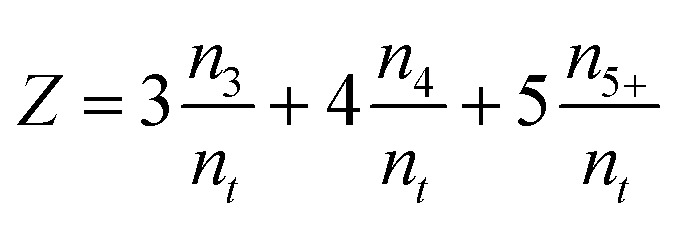
with
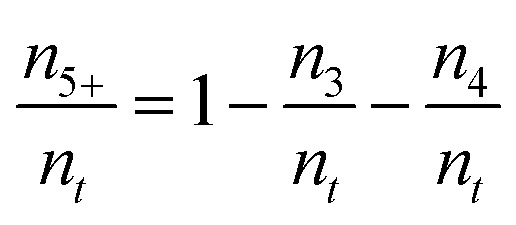
where *n*_*t*_ is the total number of junctions or node, *n*_3_ is the number of nodes with three branches connection, *n*_4_ the number of nodes with 4 connection, and *n*_5+_ the number of higher-order node with 5 or more connected branches. Therefore, the fraction of network connection can be reported by *n*_3_/*n*_*j*_, *n*_4_/*n*_*t*_ and *n*_5+_/*n*_*t*_. The main advantage of this method is the possibility of interpreting the connectivity of the pore system without separating and defining the limits of individual pores.

## 3. Results and discussion

### 3.1. PXCT under ambient conditions

**Fig. 2:**
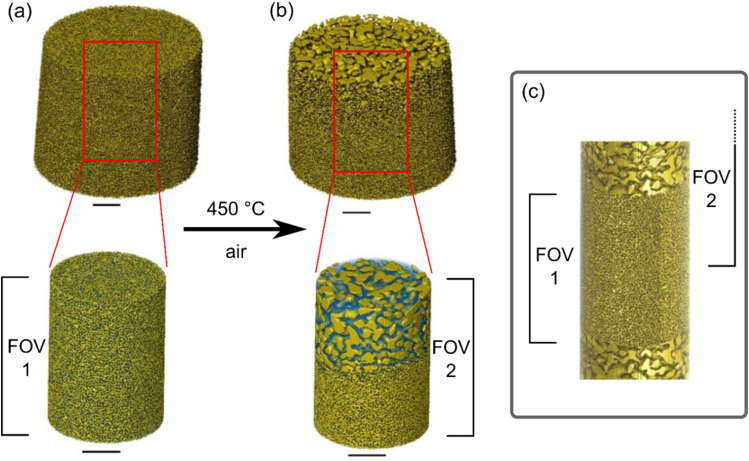
Reconstructed PXCT data showing the total np-Au (1) sample and a central sub-volume of each sample: (a) before (FOV 1) and (b) after (FOV 2) heating to 450 °C in air for *ca.* 1 hour. Note that the volumes (a) and (b) do not correspond to exactly the same sample volume but are vertically offset by *ca.* 3 µm. In particular, the areas with a coarser porosity in (b) and (c) were not included in the volume probed in (a). The volume shown in (c) was recorded in a separate scan, indicating the original FOV of the preceding scans (a) and (b). All scale bars are 1 µm.

The first np-Au sample (np-Au (1), *ca.* 5 µm diameter, 5 µm height) was initially measured *via* PXCT at ambient conditions (*ca.* 23 °C, ambient air). Fig. 2a and b shows the reconstructed tomograms before and after heating at 450 °C, with spatial resolution of 19 nm (SI, Section 5). These scans were offset vertically by *ca.* 3 micron despite moving to an identical motor position, probably due to morphological change and densification of the sample cylinder as a result of thermal annealing. As a result of this offset, we observed that the thermal annealing effects were notably not uniform throughout the sample. A larger vertical scan was therefore performed to visualize this effect in more detail (Fig. 2c). The volume represented by FOV 1 (Fig. 2a) was uniquely exposed to X-rays for *ca.* 8 hours before thermal annealing, and as a result appeared to be completely stabilized, *i.e.* no annealing was observed in any part of the volume. In neighboring vertical regions above and below FOV 1 which were not directly exposed to X-rays before annealing, partial stabilization was observed at the absolute exterior of the sample (Fig. 2b and c), while parts of the center of the sample cylinder (FOV 2) were annealed as originally expected. From Fig. 2 the maximum pore size observed for the annealed sections of the sample were visibly larger than the stable sections.

To explain this behavior, we hypothesize that the concurrence of X-ray illumination, atmospheric air, and the presence of high surface area Au sites (FOV 1) triggered a stabilization effect on the np-Au solid, so that the annealing process was significantly hindered. This effect was most notable on the area which was significantly exposed to direct X-rays (FOV 1), while neighboring regions above and below showed only a small stable zone of *ca.* 100–200 nm, possibly from a smaller radiation dose at the border of the exposed region. A possible explanation for this stabilization can be due to reduction of carbon dioxide from ambient air, leading to contamination of the gold surface with carbon. Although such an observation was not within the original experiment plan of this study, it is of significant interest for future thermal degradation experiments, having not been directly visualized previously for np-Au to the best of our knowledge. We note that this stabilization is distinct from that caused by the presence of metal oxides, which was reported previously by Wittstock *et al.* and Biener *et al.*^31,32^ Further discussion is included in Section 3.5 below.

### 3.2. PXCT under inert conditions and thermal annealing results

**Fig. 3:**
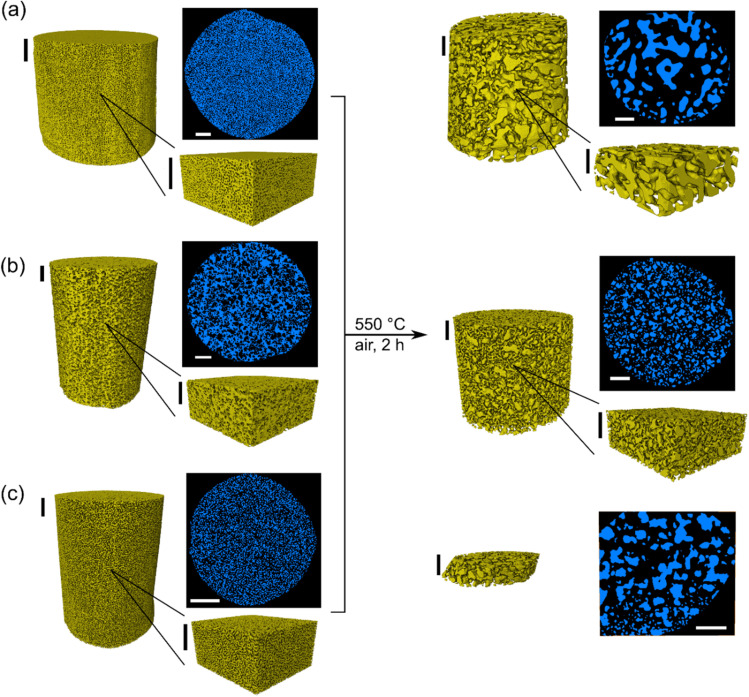
Summary of PXCT reconstructions before and after thermal annealing, showing degradation of the pore system for (a) np-Au (2), (b) CeO_*x*_/np-Au, and (c) Ce-TiO_*x*_/np-Au. Each dataset shows the total volume recorded and a typical orthographic slice to illustrate the observed pore system. Arbitrary subsections are shown for (a) and (b) but not (c) due to sample tilt which decreased the available volume which could be imaged without artefacts from the sample holder. All PXCT data were recorded under N_2_ flow. All scale bars are 1 µm.

All further experiments were performed under continuous inert N_2_ flow during PXCT measurements. Following the hypothesis of carbon deposition from ambient air, the aim was to minimize the presence of possible contamination and unwanted stabilization, allowing thermal annealing to be observed as intended. An overview of PXCT results for np-Au (2), CeO_*x*_/np-Au, and Ce-TiO_*x*_/np-Au is shown in Fig. 3a–c, respectively. The partial stabilization effect observed previously during PXCT in ambient air was not present here, therefore the entire sample responded as expected to thermal annealing. Annealing of each sample in each case can be interpreted as a densification of the solid phase, resulting in an increased diameter of larger visible mesopores (2–20 nm) and macropores (>20 nm), together with an assumed decrease in the volume of micropores present (<2 nm). All porosity figures are taken from the IUPAC definition,^53^ meaning that micropores and smaller mesopores were essentially below the resolution limit of PXCT, while larger mesopores and macropores could be observed in this study. The relevance of this resolution limit for studying thermal annealing of np-Au is discussed in Section 3.5 below. While the first annealing step at 550 °C largely retained the initial structure of each sample, the second annealing step at 750 °C caused extensive degradation and annealing leading to a largely complete loss of visible porosity. The following analysis of the pore system characteristics therefore mainly focuses on the initial sample states and those following the first annealing step at 550 °C.

### 3.3. Global porosity analysis of PXCT data

From PXCT it was possible to firstly derive basic global porosity values of the visible pore system. It should be noted that emphasis is placed here on the visible porosity only and does not account for possible pore features below the estimated spatial resolution of the tomograms. The latter can however be additionally interpreted from PXCT data by analyzing the quantitative electron density (SI, Section 4) of the solid phase, as described in our previous work.^54^ As a result of this global analysis, the percentage visible porosity and the specific surface area of the samples were calculated, as shown in Table 1.

**Table 1:** Global physical properties derived from rendering data volumes of PXCT

Sample	Temperature (°C)	Porosity^a^ (%)	Specific surface area	
			(m^2^ m^−3^)	(m^2^ g^−1^)
np-Au-(2)	20	39.6 ± 0.04	5.93 × 10^7^	3.07 ± 0.27
	550	64.3 ± 0.01	1.43 × 10^7^	0.74 ± 0.00
	750	59.5 ± 0.00	3.39 × 10^6^	0.18 ± 0.00
CeO_*x*_/np-Au	20	64.2 ± 0.01	4.58 × 10^7^	2.37 ± 0.05
	550	66.6 ± 0.01	4.11 × 10^7^	2.13 ± 0.04
	750	42.9 ± 0.00	4.68 × 10^6^	0.24 ± 0.00
Ce-TiO_*x*_/np-Au-(1)	20	64.7 ± 0.03	7.25 × 10^7^	3.76 ± 0.18
	550	63.7 ± 0.02	2.66 × 10^7^	1.38 ± 0.02
	750	63.1 ± 0.00	5.41 × 10^6^	0.28 ± 0.00
Ce-TiO_*x*_/np-Au-(2)	20	44.2 ± 0.04	6.00 × 10^7^	3.11 ± 0.26
	450	61.1 ± 0.02	4.83 × 10^7^	2.50 ± 0.15

a Accounting only for porosity within the spatial resolution limit. Error values indicate ± 1 standard deviation based on three datasets (1–3) with volume thresholding at nominal values (1), 10% above nominal values (2) and 10% below nominal values (3).

The first annealing step at 550 °C caused a decrease in specific surface area for all measured samples, with np-Au (2) showing the largest decrease (76% loss in surface area), followed by Ce-TiO_*x*_/np-Au (63% loss) and CeO_*x*_/np-Au (11% loss). A notable difference in the extent of annealing was observed for the Ce-TiO_*x*_/np-Au sample, a sample of which was studied after annealing at 450 °C or 550 °C. While the latter sample showed extensive annealing, the former was largely stable (SI, Fig. S3). The difference in resolution between the room temperature series Ce-TiO_*x*_/np-Au (2) (*ca.* 24 nm) and Ce-TiO_*x*_/np-Au (1) (*ca.* 18 nm) should be noted however, since this may explain the apparently lower initial porosity in the former case. In general, the results are consistent with our previous work and other literature studies showing stabilization of np-Au by the incorporation of (mixed) metal oxide species on the surface of the gold ligaments, as observed by electron microscopy and *in situ* 2D ptychography.^32,40,55^ However, the stabilization effect is clearly dependent not only on the presence of metal oxide species, but also on the applied temperature. The annealing was further reflected by a change in the total visible porosity, with ligament densification resulting in a large increase in porosity for np-Au (2), while the metal oxide containing samples showed relatively little change and therefore being more resistant to thermal annealing. Further discussion is included at the end of the results section. Since such global pore system analysis does not fully exploit the value of high-resolution tomography, further interpretation of the pore system was performed by skeletonization and connectivity analysis.

### 3.4. Skeleton analysis and pore network connectivity

**Fig. 4:**
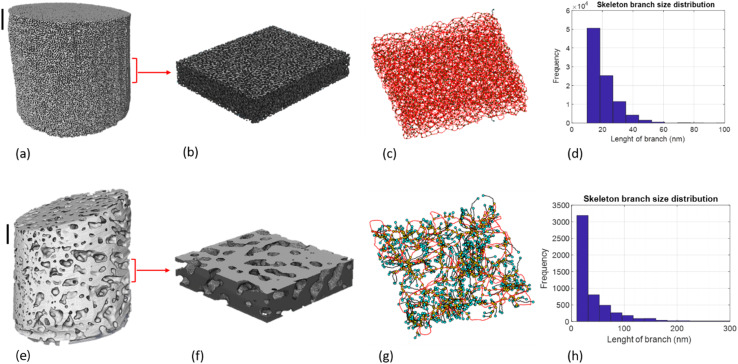
Pore volume and skeleton analysis on sub-volume of fresh np-Au (a–d) and heated sample up to 550 °C (e–h). All scale bars are 1 µm.

**Fig. 5:**
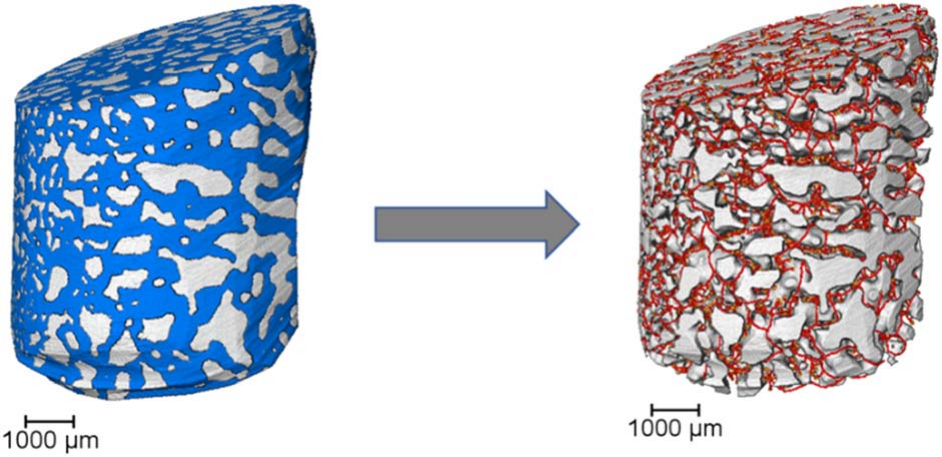
Skeletonization applied for whole image of np-Au sample after heating up to 550 °C.

An overview of the skeletonization process is shown in Fig. 4 and 5. The quantified results for skeleton analysis shown in Table 2 reveal that for np-Au (2), the total pore network length decreases dramatically after heating up to 550 °C. This confirms that the sample sintered significantly on heating, leading to collapse of the mesopore system within the spatial resolution limit of the measurement. For Ce-TiO_*x*_/np-Au (1), the decrease in network length may be anomalous, due to the limited usable scan range compared to the other samples, despite normalization to the available volume. This was due to sample tilt which decreased the available volume which could be imaged without artefacts from the sample holder. No significant changes were observed for a second sample of Ce-TiO_*x*_/np-Au which was heated up to 450 °C, showing the absence of any major degradation up to this temperature.

**Table 2:** Skeleton analysis of sample series before and after first heating step

Sample-*T* (°C)	Per volume (1 µm^3^)			Median length of branches (nm)
	Total pore network length (nm)	N. EP^a^	N. BP^b^	
np-Au-20 (2)	227	40.9	2804	13.2
np-Au-550 (2)	18	58.4	66	41.9
CeO_*x*_/np-Au-20	73	32.6	520	22.5
CeO_*x*_/np-Au-550	100	71.4	477	28.3
Ce-TiO_*x*_/np-Au (1)-20	101	0.03	996	12.5
Ce-TiO_*x*_/np-Au (1)-550	20	27.7	86	25.1
Ce-TiO_*x*_/np-Au (2)-20	132	0.99	1303	17.1
Ce-TiO_*x*_/np-Au (2)-450	135	12.8	1082	19.3

a N. EP: number of end point.

b N. BP: number of branch point (node).

Comparison between samples shows that changes in the pore network system are more considerable for pure Au than for other samples and we can conclude that the deformation of the structure in this case is severe. Again this points to stabilization of np-Au towards thermal annealing due to the inclusion of metal oxides. This is further discussed at the end of the results section. Moreover, the results show that the median length of branches after heating increased for all samples. This means that in fresh samples the length of the branches without any connections is shorter, while after heating the number of branches (or number of branch point) in the pore system decreases and the branch length increases. Overall this shows that the structure of the bed becomes less tortuous.

Pore connectivity analysis indicating the number of branches which meet at junctions are presented in Table 3. This parameter was calculated for different sub-volumes of each sample to investigate the homogeneity of the pore system and the changes induced by heating. The results show largely uniform pore connectivity for each sub-volume, indicating that the samples cylinders prepared for PXCT are representative of the parent material within the spatial resolution limit of the measurement. The pore connectivity values decreased for all samples after heating up to 550 °C, indicating the occurrence of pore sintering during heating. The pore connectivity varies only slightly over Ce-TiO_*x*_/np-Au (2) after heating up to 450 °C and CeO_*x*_/np-Au after heating up to 550 °C. The highest pore connectivity numbers were recorded for Ce-TiO_*x*_/np-Au sample, meaning that for most of the pore junctions (throats) more than four branches are connected. A severe decrease of up to 18% in pore connectivity was observed for pure Au after heating up to 550 °C. The final value of pore connectivity number for pure Au after heating is 3.15 which shows the high degree of pore system sintering during heating.

**Table 3:** Pore connectivity analysis

Sample-*T* (°C)	Pore connectivity	
	Mean value from sub volumes	Whole sample
np-Au-20 (2)	3.87 ± 007	3.84
np-Au-550 (2)	3.15 ± 006	3.15
CeO_*x*_/np-Au-20	3.49 ± 012	3.6
CeO_*x*_/np-Au-550	3.27 ± 009	3.28
Ce-TiO_*x*_/np-Au (1)-20	4.04 ± 005	4.04
Ce-TiO_*x*_/np-Au (1)-550^a^	3.40	—
Ce-TiO_*x*_/np-Au (2)-20	4.2 ± 001	4.2
Ce-TiO_*x*_/np-Au (2)-450	3.83 ± 006	3.87

a Due to constraints in the scanning process, analysis was limited to a single sub-volume of the sample.

### 3.5. Discussion of sintering process, stabilization effect, and evaluation of PXCT

The mechanism of annealing in np-Au has been discussed in literature, and like many porous materials is generally thought to involve surface diffusion by the Gibbs–Thomson effect. Essentially, strongly curved surfaces with low density and nanoscale size show larger deviation (decrease) from the ideal melting point for that substance.^56,57^ The specific adsorbates on the surface and the related gas atmosphere during annealing are therefore expected to play an important role. In this context, previous studies have shown that thermal annealing of np-Au in vacuum or inert gases shows much higher onset temperature for annealing compared to synthetic air (20% O_2_/He).^55,56^ This indicates that chemisorbed oxygen on the np-Au surface is released at elevated temperatures, leading to surface vacancies which promote annealing and ligament deformation Several studies have shown that significant annealing is linked to the desorption temperature for oxygen.^56,57^

Notably, it is unlikely that the change in stability and the unusual macroscopic stabilization effect observed here was due to the gas environment during annealing, since this was always done in ambient air in a furnace. Instead it is probable that the gas environment during tomography measurements, which combined extremely high surface area gold and high intensity X-rays, contributed to this stabilization. For measurements under flowing N_2_ the samples subsequently annealed significantly, while the stabilization effect was observed during PXCT in ambient air. Since this result is apparently contradictory to several literature studies showing decreased annealing temperature and more extensive annealing in the presence of oxygen, we reinforce the idea that it is in fact the presence of ambient air containing trace carbon in combination with X-ray exposure which appears to make a difference in improved stabilization. In a previous X-ray imaging study with synthetic air, no carbon was present during annealing, and the annealing onset temperature was notably decreased as a result.^55^

It should also be noted that while there are no known reports of this specific stabilization effect in literature to the best of our knowledge, a comparison of SEM annealing studies and X-ray imaging annealing studies reveals an interesting discrepancy. Specifically, Chen-Wiegert *et al.* used synchrotron X-ray nanotomography to study annealing of np-Au in air *ex situ*, analogous to the present work. Only minor changes in ligament size for np-Au were noted even after 1 hour at 550 °C, while lower temperatures were not directly investigated and the temperature selection criteria were not clearly stated.^58^ On the other hand, Chen *et al.* made a comparable study by sequential SEM imaging following annealing of np-Au under O_2_, inert (Ar), and (CO) atmospheres, noting more significant annealing under O_2_ already below 200 °C compared to other gases.^56^ Since both studies involve annealing of np-Au with similar structure, although with starting composition of Au_35_Ag_65_ (Chen *et al.*) and Ag_30_Au_70_ (Chen-Wiegert *et al.*), respectively, it is plausible that the increased temperatures required during the X-ray imaging study (also noted in the current work) are due to the same unexpected stabilisation effect as observed here. While we did not investigate lower annealing temperatures here, future tomography studies may explore this further by attempting to anneal under similar temperature conditions to those of Chen *et al.* This would provide further evidence of the X-ray induced stabilisation, although the exact chemical reason for this effect requires further investigation. It is also notable that the aforementioned annealing studies in CO atmosphere resulted in increased stability with higher onset temperature of 600 °C for annealing compared to both inert (400 °C) or O_2_ (200 °C) atmosphere. However, it is not clear whether this effect may be related to the macroscopic stabilisation observed here.^56^

Literature studies on np-Au functionalized with different metal oxide nanoparticles have consistently shown improved durability towards thermal annealing. At the same time, annealing studies have also been shown to decrease the size and increase the dispersion of metal oxide agglomerates within the nanoporous structure.^40^ If certain metal agglomerates are in principle contributing to blocked pores in the as-prepared samples, this behaviour may explain the general increase in total pore length for CeO_*x*_/np-Au treated at 550 °C and Ce-TiO_*x*_/np-Au treated at 450 °C, as outlined in Table 2. This is also consistent with no such effect being observed for pure np-Au, which simply undergoes thermal annealing leading to a decrease in total pore length. The reason for highest pore connectivity with a mixed Ce/Ti material probably indicate greater thermal stability compared to a single component metal oxide, although the mechanistic reason for this would require further spectroscopic or sorption investigations, in a similar manner to Watanabe *et al.* for mixed TiO_2_–CeO_2_ (without np-Au),^59^ which are outside the current scope.

Quantitative analysis of tomographic data, for example with PXCT, is only possible according to the visible features of interest, which may not correspond to the total features of interest. In this case, only large mesopores and macropores were visible due to the resolution limit of the measurements, which probably does not reflect the total porosity in the sample. Specifically, np-Au is known to contain micropores, as observed by previous studies based on FIB-SEM and electron tomography, which were not resolvable here.^19^ When a quantitative assessment of the entire pore system is desired using 3D imaging, this generally cannot be obtained by any individual imaging method but rather benefits from a combination. This was shown in our previous work on hierarchically porous Ni/Al_2_O_3_ catalysts,^54^ and on np-Au samples.^19^ For example, previous complementary electron tomography, FIB-SEM, and PXCT measurements on similar np-Au samples revealed total porosity of 60% for PXCT and 52% for electron tomography, which is similar despite very different sample volumes (300 µm^3^ and 1 µm^3^, respectively) and spatial resolution (23 nm and 1–3 nm, respectively). FIB-SEM on the other hand showed total porosity of 30–39% depending on the sub-volume. This is most probably due to imaging artefacts which can occur when FIB-SEM is performed on large pore features in the absence of any contrast or filler material, known as “shine-through” artefacts. Notably, Larsson *et al.* also studied np-Au prepared by similar methods using transmission X-ray nanotomography, achieving spatial resolution around 63 nm.^29^ Despite the difference in resolution compared to our previous study (23 nm) and the current work (20 nm), in all three cases the visible porosity was around 60%, and in the Larsson study and the current work, the pore connectivity for the fresh np-Au sample had an approximate value of 4. This strongly suggests that np-Au, at least in the freshly prepared state, has a relatively uniform distribution of pore sizes at least in the range of around 3–100 nm diameter. This indicates that provided measurements are obtained with relatively similar spatial resolution as in the current work, that a representative analysis of pore system changes is feasible. However, it should be noted that a detailed characterization of the total pore system was not in scope during this work, rather the relative changes on thermal annealing were of interest.

The overall value of the PXCT method can be summarised as the ability to assess a relatively large field of view with relatively high spatial resolution, compared to similar 3D nanotomography methods. This arguably provides more physically representative data compared to investigating only with electron tomography (limited field of view and sample size) or FIB-SEM tomography (completely destructive), as shown previously for np-Au. It is also reasonable to suggest that the macroscopic stabilization effect observed here, which required *in situ* heating in defined gas environments, could only be both observed and quantified using PXCT or similar hard X-ray nanotomography methods. The spatial resolution limits were more than sufficient to observe this effect and therefore do not negatively impact the goal of this work, which was the observation of annealing processes on large sample volumes over extended length scales.

## Conclusions

PXCT was used to visualize stochastic pore systems in np-Au at spatial resolutions of *ca.* 10–20 nm. Among currently available hard X-ray nanotomography methods, PXCT offers an optimum field of view and spatial resolution for visualizing such features. Thermal annealing effects were found to be less prevalent in terms of specific surface area loss for np-Au with metal oxide additives present, determined by visualization of the pore networks over an extended length scale of several microns. In addition, the unexpected stabilization towards thermal annealing of samples exposed to X-rays in ambient air was definitively observed and may be interesting for future studies on material stability. PXCT was not only able to visualize the 3D morphology of np-Au materials before and after thermal annealing, but also to quantitatively analyze pore structures. The data obtained are sufficient to build detailed models of pore structures ranging from larger mesopores to macropores through established image processing methods. Pore system analysis was performed through skeletonization, therefore extending beyond common porosity and surface area values. This in turn allows a detailed mathematical description of porosity, including the presence of blocked or inaccessible nodes (where these may occur), the connectivity and branching nature of pores, and the distribution of pores within 3D space. The data presented here represent an effective approach which can be extended to accurately describe pore system composition in other relevant samples, such as technical or industrial catalysts or sorbent materials. Here np-Au is a useful test object for maximum resolution imaging due to its stochastic pore structure and excellent scattering contrast between noble metal and voids, as well as porosity from the micro to macropore range. Particularly in the context of current and upcoming fourth generation synchrotron light sources, np-Au is a suitable choice for possible performance comparison between next generation hard X-ray nanotomography instruments.

## Conflicts of interest

There are no conflicts to declare.

## Supplementary Material

NA-OLF-D5NA00561B-s001

## Data Availability

Data for this article, including reconstructed ptychographic tomography volumes, are available at KITOpen, the open access repository of Karlsruhe Institute of Technology at https://doi.org/10.35097/89wnns8dbfgffjzt. Additional data supporting this article have been included as part of the supplementary information (SI). Supplementary information: tomography image processing, skeletonization procedure, additional images of the stabilization effect, electron density analysis, ptychographic tomography scan parameters and resolution estimation. See DOI: https://doi.org/10.1039/d5na00561b.
