# Supplementary material for: Hard X-ray nanotomography reveals anomalous and expected thermal coarsening behaviour of nanoporous gold
Source: Nanoscale Adv. 2025 Nov 20;8(2):662–72. doi: 10.1039/d5na00561b (PMC12699826; doi:10.1039/d5na00561b)
Supplement: NA-008-D5NA00561B-s001 [file NA-008-D5NA00561B-s001.pdf]

Supplementary Information (SI) for Nanoscale Advances.
This journal is © The Royal Society of Chemistry 2025

Electronic Supporting Information

Hard X-ray nanotomography reveals anomalous and expected thermal coarsening behaviour of nanoporous gold

Reihaneh Pashminehazar[1,2], Yakub Fam[1,†], Ana Diaz[3], Mirko Holler[3], Michal Kronenberg[3,‡], Johannes Ihli[3], Jan-Dierk Grunwaldt[1,2,*], Thomas L. Sheppard[1,⊥*]

[1]Institute for Chemical Technology and Polymer Chemistry, Karlsruhe Institute of Technology, Engesserstrasse 20, 76131 Karlsruhe, Germany

[2]Institute of Catalysis Research and Technology, Karlsruhe Institute of Technology, Hermann-von-Helmholtz Platz 1, 76344 Eggenstein-Leopoldshafen, Germany

[3]PSI Center for Photon Science, Paul Scherrer Institut, Forschungsstrasse 111, 5232 Villigen PSI, Switzerland

[†]Current address: Thermo Fisher Scientific, Brno, Czechia

[‡]Current address: Carl Zeiss SMT, 22, Carl-Zeiss-Straße, 73447, Oberkochen, Germany

[⊥]Current address: Institute of Chemical Technologies and Analytics, TU Wien, Getreidemarkt 9, 1060 Vienna, Austria

Corresponding author: thomas.sheppard@tuwien.ac.at, grunwaldt@kit.edu

1. Tomography Image Processing

Here we describe the processing of ptychographic X-ray computed tomography (PXCT) 3D datasets obtained as described in the main text. The first step of preparing the gray value image for image processing is filtering which is a technique for smoothing (denoising) or enhancing an image. The median filter is one of the well-known order-statistic filters due to its good performance and widely used in image processing to improve the results of later processing. However, it doesn't function properly for a sample with high variation in gray value and tends to blur edges in images. Therefore, for highly porous samples an edge-preserving smoothing technique was used to smooth away noise and enhance the image while preserving edges (Figure S*1*). Based on Otsu's thresholding method [1] a binary image was created: the value 1 (white) was assigned to all the pixels with intensity higher than the given gray tone value (threshold), while the value 0 (black) was assigned to the other pixels, i.e. pores and background.

Morphological closing is a useful method for filling the small pores of the object in an image. This function consists of a dilation followed by an erosion operation with the same structuring element. Therefore, it is less destructive than other morphological operations and it preserves the original size, shape and convexity of the analyzed structure. The volume of small pores in the sample can be defined very well with this method but for the images with sintered pores or large pores, the closing method is not applicable.

It is not possible to fill a large pore without an undesired deformation of the exterior surface of the object. Therefore, for such samples a convex hull method was used to distinguish between background air and pore. The convex hull is the smallest convex polygon or polyhedron that completely circumscribes an object. For this purpose, the convex hull function of Avizo 9.7 software (ThermoFisher Scientific, USA) was used and applied over the 3D image slice by slice. After this procedure, the basic characterization such as porosity and surface area can be exported and calculated. All the 3D volume renderings of the entire sample and extracted region of interest were performed using the Avizo software.

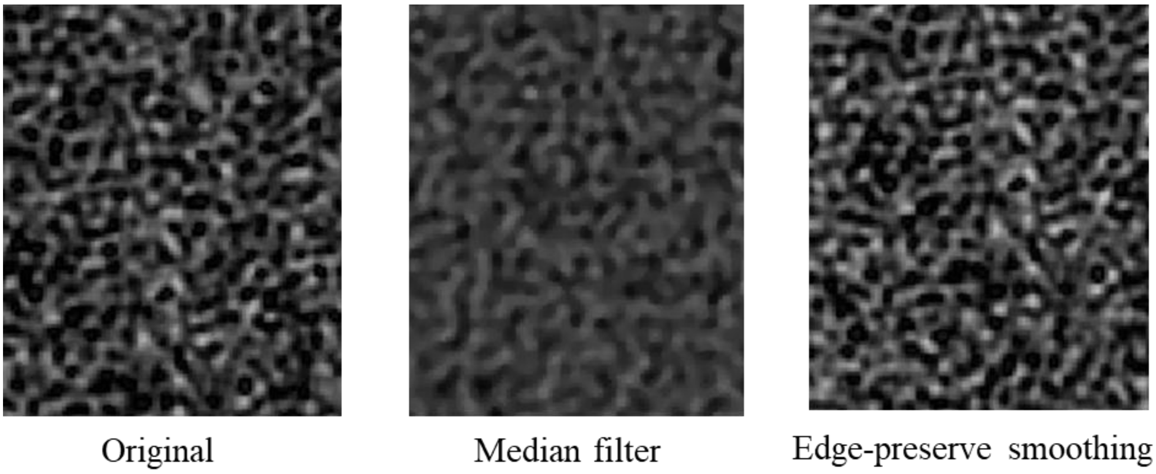

Figure S1. Applying filtering for denoising and enhancing a typical image of np-Au

2. Skeletonization and Skeleton Analysis

Skeletons have several different mathematical definitions in the technical literature, and there are many different algorithms for computing them. In this research the Matlab function *bwskel* is used to generate skeleton structure of the pore system. This function uses the medial axis transform and more details are available in the literature [2]. The input value for this function should be 2D or 3D binary image in which foreground pixels are 1 (white) and the background ones are 0 (black). To make the binary image suitable for pore skeletonization, the complement of the image should be taken in a way that the pore is white and the material is black. Finally, a pruning algorithm was used to remove unwanted and small skeleton branches in order to get a clean skeleton representation.

Node (branch points-Bp) are the voxels at the junction where multiple branches meet (voxels have more than 2 neighbors) and end points (Ep) are voxels at the ends of branches (voxels have less than 2 neighbors). The simple clarification is represented in Fig. S2.

The skeleton analysis was applied to different sub-volumes of each sample for comparison. To run skeleton algorithms for the entire sample image, we used another workstation. This system is equipped with an Intel® XeonR CPU E5-2643 v4 operating

at 3.4 GHz, paired with 256 GB of RAM. This choice was made to efficiently handle the larger volume of data.

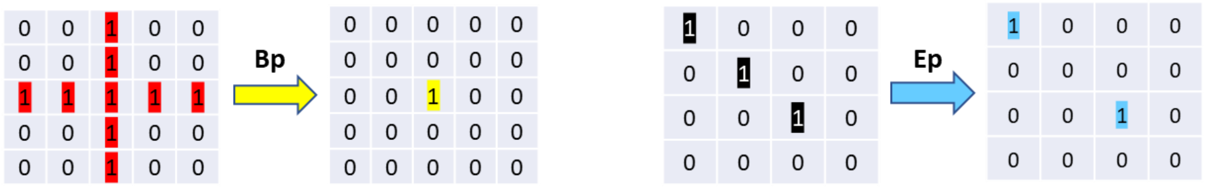

Figure S2. Methodology for defining the branch point (Bp) and end point (Ep) during skeletonisation

3. Additional data on stabilization effect in ambient air

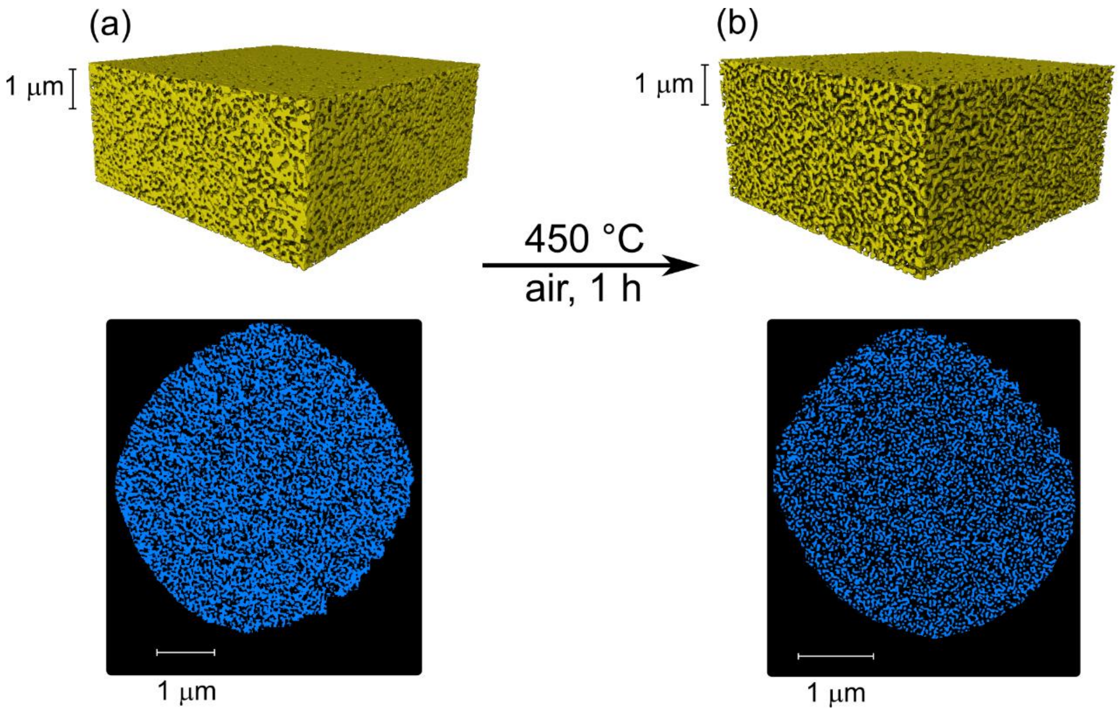

Figure S3. PXCT subsection of TiO_2-npAu before and after annealing at 450°C. PXCT measurements performed in ambient air, underlining the stabilisation effect and minimal observable annealing.

4. Electron density analysis of samples after annealing

Figure S4 shows the electron density values for three selected samples, where the individual electron density from all pixels is counted to generate histograms. An explanation of how to obtain electron density values from reconstructed ptychography images can be found in previous work. [3]

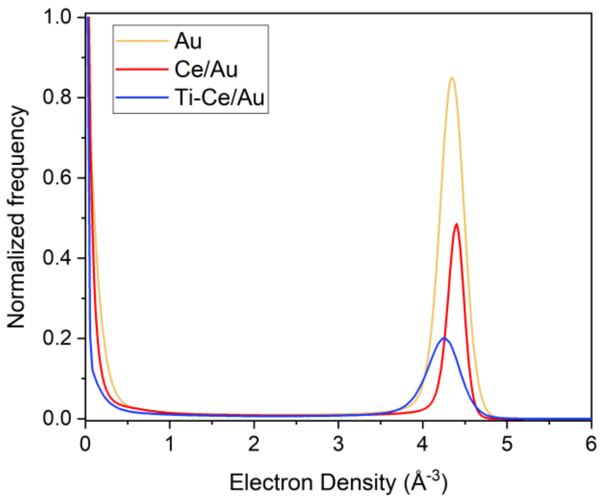

Figure S4. Electron density of sample after heating up to 750 °C

Figure S5 shows the averaged electron density values from all pixels in each horizontal slice, with error bars indicating +/- 1 standard deviation. While this eliminates the individual 3D spatially-resolved data from each pixel, it shows that the data is consistent over a large volume of space. In other words, it shows the homogeneity of the sample in the vertical direction and confirms that the average electron density shown in Figure S4 is replicated throughout the entire volume. Significant deviation would point to non-homogeneous sample structure (which would make data interpretation difficult) or error/instability in the ptychographic and/or tomographic image reconstruction, or other measurement artefacts.

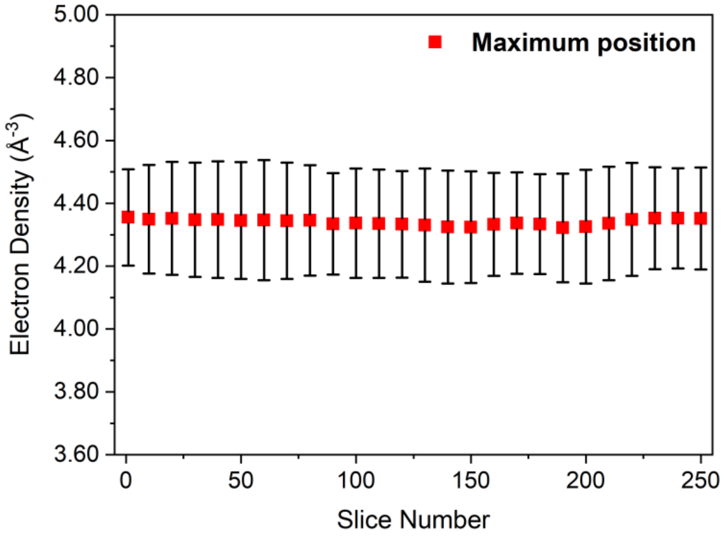

Figure S5. Electron density distribution of gold at different slices of npAu sample heated up to 750 °C, showing consistent composition and structure of the entire volume.

5. Resolution estimation of PXCT data by Fourier Shell Correlation

Fourier shell correlation (FSC) was used to estimate the resolution of the tomograms. This was performed by dividing one complete tomogram into two subtomograms with equal angular spacing, then reconstructing the tomograms individually. The two reconstructed subtomograms were then compared for spatial similarities, to estimate the effective 3D spatial resolution of the full tomogram according to the ½-bit criterion [4].

Table S1: List of measurements performed in this study. (1) and (2) indicates the first and second sample of the same type. RT denotes measurements performed at room temperature and N2 means that the sample was under a N_2 gas flow during the measurement

Meas. number (#)	Sample name	Sample condition	Measurement condition
1	np-Au (1)	before treatment	in air, RT
2	np-Au (1)	after 450°C treatment	in air, RT
3	np-Au (2)	before any treatment	in N2, RT
4	np-Au (2)	after 550°C treatment	in N2, RT
5	np-Au (2)	after 750°C treatment	in N2, RT
6	CeO_x/np-Au	before any treatment	in N2, RT
7	CeO_x/np-Au	after 550°C treatment	in N2, RT
8	CeO_x/np-Au	after 750°C treatment	in N2, RT
9	Ce-TiO_x/np-Au (1)	before any treatment	in N2, RT
10	Ce-TiO_x/np-Au (1)	after 550°C treatment	in N2, RT
11	Ce-TiO_x/np-Au (1)	after 750°C treatment	in N2, RT
12	Ce-TiO_x/np-Au (2)	before any treatment	in N2, RT
13	Ce-TiO_x/np-Au (2)	after 450°C treatment	in N2, RT

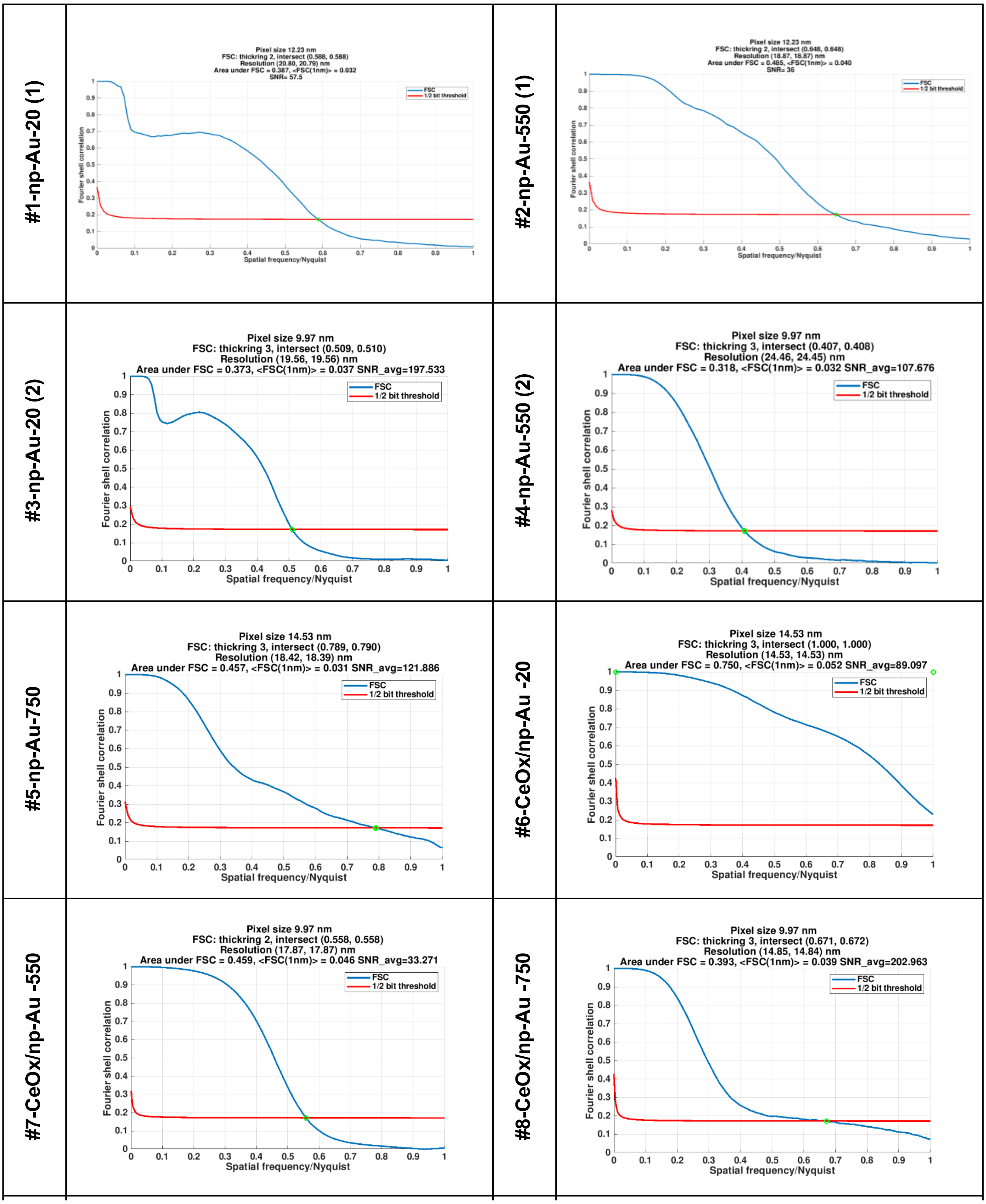

Figure S6. FSC analysis of PXCT data from measurements at cSAXS

#9-Ce-TiOx/np-Au(1)-20

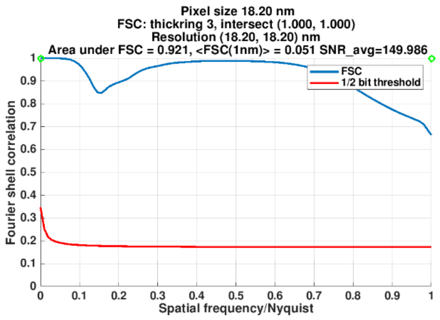

#10-Ce-TiOx/np-Au (1)-550

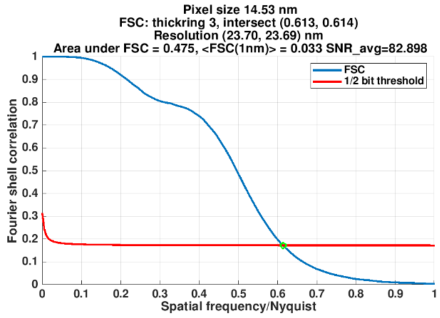

#11-Ce-TiOx/np-Au (1)-750

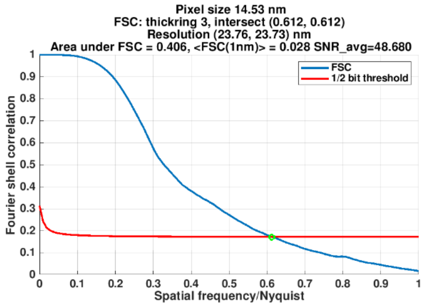

#12-Ce-TiOx/np-Au (2)-20

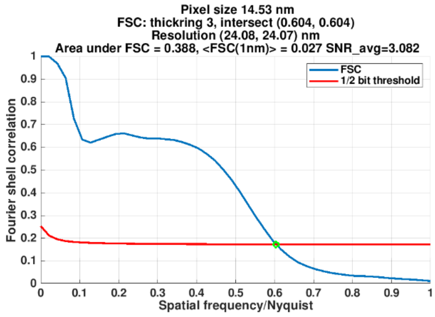

#13-Ce-TiOx/np-Au (2)-450

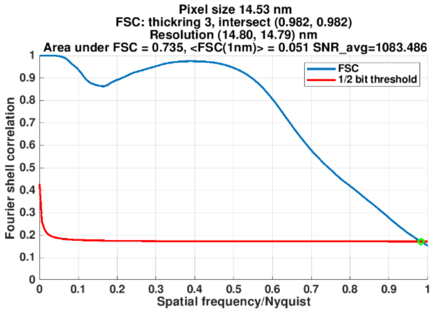

Figure S6 (continued). FSC analysis of PXCT data from measurements at cSAXS

Table S2: Setup parameters for each of the measurements. Measurement numbers are denoted by # (see table S1); E: photon energy, FZP: Fresnel zone plate (60 nm outer-most zone width), ø: diameter, beam size: approximate beam size on sample

#	E (keV)	FZP ø (μm)	FZP focal distance (mm)	sample-focus distance (mm)	beam size (μm)	detector pixel size (μm)	sample-detector distance(m)
1	6.200	170	51.0	0.8	2.7	172	7.362
2	6.200	170	51.0	0.8	2.7	172	7.362
3	6.200	220	66.0	0.9	3.0	75	5.232
4	6.200	220	66.0	0.9	3.0	75	5.232
5	6.200	220	66.0	0.9	3.0	75	5.232
6	6.200	220	66.0	0.9	3.0	75	5.232
7	6.200	220	66.0	0.9	3.0	75	5.232
8	6.200	220	66.0	0.9	3.0	75	5.232
9	4.951	220	48.9	0.9	4.0	75	5.232
10	6.200	220	66.0	0.9	3.0	75	5.232
11	6.200	220	66.0	0.9	3.0	75	5.232
12	6.200	220	66.0	0.9	3.0	75	5.232
13	6.200	220	66.0	0.9	3.0	75	5.232

Table S3: data acquisition parameters for each of the measurements. Measurement numbers are denoted by # (see table S1); FOV: field of view, step: average step size in ptychographic scans, ct: exposure time at each scanning position; N_{proj}: number of projections; T: total acquisition time including dead time during movement of stages; dose: estimated dose absorbed by the specimen.

#	FOVx (μm)	FOVy (μm)	step (μm)	ct (s)	N_{proj}	T (h)	dose (Gy)
1	8	5	0.5	0.1	1000	5.5	5.0E+09
2	8	5	0.5	0.1	757	9	4.0E+09
3	8	5	0.7	0.05	650	2	8.8E+08
4	10	5	0.7	0.05	700	2.5	9.5E+08
5	10	6	0.7	0.05	700	2.5	9.5E+08
6	10	10	0.7	0.05	1400	8	1.9E+09
7	14	12	0.7	0.05	850	7.5	1.2E+09
8	10	10	0.7	0.05	1400	7	1.9E+09
9	8	8	0.5	0.1	1000	10	1.4E+09
10	20	8	0.8	0.05	700	5	1.2E+09
11	10	5	0.7	0.05	700	2.5	9.5E+08
12	8	2	0.7	0.1	700	1.5	1.9E+09
13	10	10	0.7	0.1	700	5.5	1.9E+09

Table S4: Data processing parameters. Binning: whether detector data were averaged in 2x2 pixels for ptychographic reconstruction. “Ptycho method” denotes the method used for ptychographic reconstruction: difference map (DM) [5], maximum likelihood optimization (ML) [6], or MLc,a generalized iterative least-squares solver implemented in GPU [7].The numbers indicate the number of iterations used with each algorithm. N_x and N_y denote the number of detector pixels used to crop the diffraction patterns along the horizontal and vertical directions; p: reconstructed pixel size, where an asterisk indicates that the images where upsampled along the vertical direction to result in an isotropic pixel size. The term SART indicated whether the tomographic reconstruction was optimized with 20 iterations of the simultaneous algebraic reconstruction technique.

#	binning	ptycho method	N_x	N_y	p (nm)	SART	resolution in 3D (nm)
1	no	DM 180 + ML 300	700	700	12.23	no	20.8
2	no	DM 180 + ML 300	700	700	12.23	no	18.9
3	yes	MLc 300	1400	960	9.97*	20 iter	19.6
4	yes	MLc 300	1400	960	9.97*	20 iter	24.4
5	yes	DM 200 + ML 200	960	960	14.53	20 iter	18.4
6	yes	DM 200 + ML 200	960	960	14.53	20 iter	14.5
7	yes	MLc 300	1400	960	9.97*	20 iter	17.9
8	yes	MLc 300	1400	960	9.97*	20 iter	14.85
9	yes	DM 200 + ML 200	960	960	18.2	20 iter	18.2
10	yes	DM 200 + ML 200	960	960	14.53	20 iter	23.7
11	yes	DM 200 + ML 200	960	960	14.53	20 iter	23.8
12	yes	DM 200 + ML 200	960	960	14.53	20 iter	24.1
13	yes	DM 200 + ML 200	960	960	14.53	20 iter	14.8

References

[1] N. Otsu, *IEEE Transactions on Systems, Man, and Cybernetics* **1979**, 9, 62-66. https://doi.org/10.1109/TSMC.1979.4310076

[2] T.C. Lee, R.L. Kashyap, C.N. Chu, *CVGIP: Graphical Models and Image Processing* **1994**, 56, 462-478. https://doi.org/10.1006/cgip.1994.1042

[3] A. Diaz, P. Trtik, M. Guizar-Sicairos, A. Menzel, P. Thibault, O. Bunk, *Physical Review B.* **2012**, 85, 020104. https://doi.org/10.1103/PhysRevB.85.020104

[4] M. van Heel, M. Schatz, *Journal of Structural Biology* **2005**, 151, 250-262. https://doi.org/10.1016/j.jsb.2005.05.009

[5] P. Thibault, M. Dierolf, O. Bunk, A. Menzel, F. Pfeiffer, *Ultramicroscopy* **2009**, 109, 338-343. https://doi.org/10.1016/j.ultramic.2008.12.011

[6] P. Thibault, M. Guizar-Sicairos,. New Journal of Physics **2012**, 14, 063004. https://dx.doi.org/10.1088/1367-2630/14/6/063004

[7] M. Odstrčil, A. Menzel, M. Guizar-Sicairos, Optics Express **2018**, 26, 3108-3123. https://doi.org/10.1364/OE.26.003108
